# Latitudinal Gradient of UV Attenuation Along the Highly Transparent Red Sea Basin

**DOI:** 10.1111/php.13112

**Published:** 2019-06-05

**Authors:** Sebastian Overmans, Susana Agustí

**Affiliations:** ^1^ King Abdullah University of Science and Technology (KAUST) Red Sea Research Center (RSRC) Thuwal 23955‐6900 Saudi Arabia

## Abstract

The tropical and subtropical oceans experience intense incident ultraviolet radiation (280–400 nm) while their water columns are thought to be highly transparent. This combination represents a high potential for harmful effects on organisms, yet only few reports on the UV penetration properties of oligotrophic tropical waters exist. Here, we present the pattern of UV attenuation over a wide latitudinal range of the oligotrophic Red Sea. We recorded spectroradiometer profiles of PAR and UV, together with chlorophyll‐*a* (Chl‐*a*) and light absorption by chromophoric dissolved organic matter (CDOM) to determine the contribution of phytoplankton and CDOM toward UV attenuation. Transparency to UV exhibited a distinct latitudinal gradient, with the lowest and highest diffuse attenuation coefficients at 313 nm (*K*_d_ (313)) of 0.130 m^−1^ and 0.357 m^−1^ observed at the northern coast off Duba, and in the south close to the Farasan islands, respectively. Phytoplankton and CDOM both modulated UV attenuation, but CDOM was found to be the key driver despite the lack of riverine inputs. We confirm that ultraviolet radiation can reach deeper into the Red Sea than previously described, which means its potential to act as a stressor and selective driver for Red Sea organisms may have been underestimated to date.

## Graphical abstract

The optical properties of the oligotrophic and highly transparent Red Sea represented by the downwelling diffuse attenuation coefficients (*K*_d_) for the integrated PAR (400–700 nm), UV‐A (320–400 nm) and UV‐B spectra (280–320 nm). PAR and UV‐B were measured during four Red Sea cruises between October 2016 and March 2018, while the UV‐A properties were determined as part of two cruises in August 2017 and March 2018.

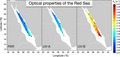

## Introduction

The intensity of ultraviolet (UV) radiation reaching the ocean surface is dependent on various factors, including latitude, season and atmospheric conditions like cloud cover, dust and tropospheric ozone 1. Upon reaching the water surface, the intensity and spectral characteristics of UV in the water column are determined by the incident irradiance, the conditions of the ambient light field (e.g. solar angle and wave action) and the concentration of UV‐absorbing and UV‐scattering substances in the water 2.

Two of the main components that modulate UV attenuation in the water column are phytoplankton and chromophoric dissolved organic matter (CDOM) 3. In the Red Sea, both components are generally found at very low concentrations. Due to the arid climatic conditions, no major river catchments flow into the Red Sea; hence, only minor amounts of nutrient‐rich terrigenous material are added via watercourses 4. Consequently, the Red Sea is one of the most oligotrophic seas in the world with a very low phytoplankton biomass (<0.5 mg Chl‐*a* m^−3^) 5, 6, 7, 8 that is mostly dominated by picophytoplankton 9, 10, 11. The only exception to this is the southern Red Sea, where phytoplanktonic biomass can reach up to 4 mg Chl‐*a* m^−3^ during blooms, and microphytoplankton such as diatoms may account for more than half of the total phytoplankton pool in terms of biomass 6, 12. In the northern Red Sea, an intense and stable water stratification occurs during the summer that inhibits the replenishment of nutrients in the surface layers, resulting in chlorophyll‐*a* (Chl‐*a*) concentrations as low as 0.05 mg Chl‐*a* m^−3^ 6.

Chromophoric dissolved organic matter (CDOM) is a chemically complex constituent of the total dissolved organic matter (DOM) that absorbs in the visible spectrum and represents the primary attenuator of UV radiation 13, 14, thereby protecting organisms in the upper euphotic zone from harmful UV 15. The chemical nature and dynamics of CDOM are highly complex and, for the most part, not well understood 16, 17, 18. However, aromatic amino acids 19, lignin phenols, and humic and fulvic acids have been identified as contributors to the CDOM pool 20, 21. Close to shore, the bulk of the CDOM pool is often allochthonous in nature and originates from terrestrial sources, rivers and coastal ecosystems such as salt marshes, seagrass meadows and mangrove forests 22, 23, 24, 25, 26, 27, 28, 29. In the open ocean, however, the primary source of CDOM is autochthonous material, which is generated by phytoplankton and heterotrophic microbial processes 13, 30, 31, 32.

Several studies have described the contribution of CDOM toward UV attenuation in the water columns of seas and oceans around the world 33, 34, 35, 36, 37, 38. However, the few existing studies on absorption by CDOM performed in the Red Sea mostly report on the role of DOM in attenuating the wavelengths of the photosynthetically active radiation (PAR, 400–700 nm) 39 or UV‐A (320–400 nm) spectra 40. These studies agree that the concentration of CDOM in the Red Sea is relatively low in a global context, due to negligible inputs from fluvial and other allochthonous land sources as well as strong photobleaching.

The parameter commonly used to define water transparency is the downwelling diffuse attenuation coefficient (*K*_d_ (*λ*)), which describes the fractional rate of decay of downwelling irradiance with depth 3. Values of *K*_d_ range widely across several orders of magnitude, from highly transparent oceanic waters to turbid coastal waters. For example, *K*_d_ (305 nm) values as low as 0.08 m^−1^ have been reported for the South Pacific Gyre, which contains some of the most optically transparent water to UV‐B globally 41, 42, while Kuwahara *et al*. 43 calculated *K*_d_ (305 nm) as high as 2.08 m^−1^ for coastal waters around the Malaysian peninsula. Other high *K*_d_ (305 nm) values have been measured in coastal waters and lagoons around the world, including southern Spain (0.58 m^−1^), Japan (1.25 m^−1^), the Great Barrier Reef (0.65 m^−1^) and the Gulf of Mexico (0.77 m^−1^) 44, 45, 46, 47.

Though the Red Sea is not oceanic by definition, it shares several characteristics with open ocean waters such as consistently low concentrations of Chl‐*a* and only minor amounts of CDOM in surface waters. Consequently, the Red Sea is thought to be among the optically transparent bodies of water worldwide 3, 8, 48. While most of the existing bio‐optical studies investigated the attenuation coefficient of the Red Sea in relation to PAR 8, 11, 39, similarly high transparency has been reported for the UV spectrum. A comprehensive study by Dishon *et al*. 49 found that the 1% attenuation depth of UV‐B (300–320 nm) varied between 19 m (*K*_d_ (UV‐B): ~ 0.24 m^−1^) and 29 m (*K*_d_ (UV‐B): ~ 0.16 m^−1^) in the Gulf of Aqaba, with the highest and lowest UV‐B transparencies measured in summer (Aug–Sep) and spring (Mar–Apr), respectively.

However, studies reporting the optical properties (particularly UV transparency) of the Red Sea are scarce and have been predominantly carried out in the far north and the Gulf of Aqaba 8, 49, 50, 51. Hence, there is a great need to study if and how the UV‐A and UV‐B attenuation properties vary, particularly in the central and southern Red Sea, since those areas experience more intense incident UV and higher sea surface temperatures (SSTs) than the Gulf of Aqaba 52, 53. High SST can exacerbate the damaging effects of UV on marine biota, including key species such as scleractinian corals and other reef organisms 54, 55, 56, 57, 58, which are of particular importance to the Red Sea 59, 60.

The goal of this study was to quantify geographical UV attenuation variability in the Red Sea, extending existing data of UV‐A and UV‐B attenuation to cover a wider latitudinal range of the basin. A further aim was to determine the extent to which UV attenuation is modulated by Chl‐*a* and absorption by CDOM (*a*_CDOM_). The present study provides a comprehensive overview of the geographical variability in UV attenuation (UV‐A and UV‐B), which can be used to determine the biologically effective optical depth and allows comparing the UV properties of the Red Sea with those of other water bodies around the world.

## Materials and methods

### *Study sites and spectroradiometer measurements*

A total of 22 spectroradiometer profiles were recorded across the Red Sea during the *Threats* and *Dust* research cruises on board R/V Thuwal in October and November/December 2016, respectively, and as part of two Center Competitive Fund (CCF) cruises in August 2017 (*CCF Summer* cruise) and March 2018 (*CCF Winter* cruise) on board R/V Al Azizi (Table 1).

**Table 1:** Sampling dates, locations and water column characteristics of the stations sampled during the cruises

Cruise	Date	Station name	Coordinates		SST (°C)	Salinity (PSU)	Chl‐*a* (mg m^−3^)	MLD (m)	*S*_275–295_ (nm^−1^)	*S*_350–400_ (nm^−1^)	*S*_R_
			Lat.	Long.							
Threats Cruise	03.10.2016	Rabigh	22.575	38.648	31.11	39.40	0.16	13	N/A	N/A	N/A
	04.10.2016	South Umluj	24.497	37.044	29.45	39.88	0.16	13	0.044	0.016	2.74
	06.10.2016	Al Wajh	26.140	36.308	29.23	39.99	0.15	25	0.039	0.012	3.38
	07.10.2016	South Yanbu	23.785	37.804	30.70	39.68	0.15	24	0.045	0.015	3.11
	08.10.2016	KAUST	22.267	38.788	30.95	39.44	0.23	22	0.046	0.011	4.09
Dust Cruise	22.11.2016	Al Lith	19.937	39.410	30.77	38.99	0.35	38	0.040	0.015	2.76
	23.11.2016	South Farasan Banks	17.736	40.422	30.13	38.33	0.46	48	0.030	0.016	1.89
	24.11.2016	Farasan Islands	17.080	41.461	30.37	38.52	0.79	41	0.028	0.014	1.97
	25.11.2016	North Farasan Islands	17.326	41.389	30.70	39.23	0.83	51	0.028	0.015	1.86
	29.11.2016	AL Wajh	25.935	36.127	28.26	39.88	0.32	72	0.019	0.011	1.69
	30.11.2016	North Yanbu	24.088	37.430	28.49	39.59	0.35	39	0.037	0.013	2.78
	01.12.2016	Rabigh	22.810	38.406	27.34	39.87	0.31	23	0.038	0.013	3.05
CCF Summer Cruise	02.08.2017	KAUST	22.276	38.788	30.67	39.19	0.14	12	N/A	N/A	N/A
	05.08.2017	Duba	27.300	34.826	29.23	40.23	0.07	22	N/A	N/A	N/A
	07.08.2017	Al Wajh	25.323	36.781	29.25	40.21	0.12	15	N/A	N/A	N/A
	14.08.2017	North Farasan Islands	17.355	40.425	32.39	38.36	0.14	11	N/A	N/A	N/A
	16.08.2017	South Jeddah	21.209	38.318	31.39	39.32	0.09	27	N/A	N/A	N/A
CCF Winter Cruise	16.03.2018	South Jeddah	21.209	38.318	26.85	38.56	0.13	28	0.030	0.018	1.69
	17.03.2018	Al Lith	19.667	39.000	27.25	N/A	0.17	36^a^	0.032	0.018	1.76
	18.03.2018	Farasan Banks	18.667	39.800	27.45	N/A	0.18	38^a^	0.040	0.019	2.10
	19.03.2018	North Farasan Islands	17.355	40.425	27.58	N/A	0.19	22^a^	0.040	0.019	2.11
	21.03.2018	KAUST	22.230	38.788	26.74	N/A	0.13	38^a^	0.037	0.018	2.13

SST = sea surface temperature, MLD = mixed layer depth, S_*λ*a–*λ*b_ = *a*_CDOM_ spectral slope in the range *λ*a–*λ*b, *S*_R_ = slope ratio (*S*_275–295_ : *S*_350–400_).

a Mixed layer depth was calculated using Δ*T* = 0.2°C instead of Δ*σθ* = 0.05 kg m^−3^.

In 2016, downwelling irradiance (*E*_d_) depth profiles at three monochromatic wavebands in the UV‐B spectral domain (305, 313, 320 nm), as well as the integrated PAR spectrum (400–700 nm), were recorded with a BIC Compact Profiling Radiometer (Biospherical Instruments, San Diego, USA). For the cruises in 2017 and 2018, a PUV‐2500 Profiling UV Radiometer (Biospherical Instruments, San Diego, USA) was used to measure downwelling irradiance (*E*_d_) for individual UV‐B (305, 313, 320 nm) and UV‐A (340, 380, 395 nm) wavelengths as well as the integrated PAR spectrum (400–700 nm). Before each measurement, an instrument dark correction was performed for the depth and optical channels. The boat was maneuvered relative to the sun to insure the hull did not cast a shadow over the instrument, and the radiometer was deployed over a pulley ~ 3 m astern using an A‐frame at a speed of approximately 0.4 m s^−1^. Because the radiometer was equipped with a pressure sensor, irradiances were recorded continuously during the profiles, generating five data points per second (5 Hz). All profiles were recorded around midday (11 am–1 pm).

### Chlorophyll‐a and CDOM analysis

As part of the *Threats*,* Dust* and *CCF Winter* research cruises, seawater samples were collected from 5 m depth in Niskin bottles attached to a CTD rosette system for quantitative analysis of Chl‐*a* and CDOM. To measure the Chl‐*a* concentration, 300 mL of seawater was filtered through a Whatman GF/F (0.7 *μ*m); the filter was transferred into a 15 mL centrifuge tube and stored at −20°C until further processing. To extract Chl‐*a*, 7 mL of 90% acetone was added to each 15 mL tube, and the samples were kept in the dark at 4°C. After 24 h, the samples were centrifuged (3000 rpm) at 18°C for 15 min. Chl‐*a* in the extract was analyzed using the nonacidification method on a Trilogy Fluorometer equipped with a Chl NA module (Turner Designs, San Jose, USA).

For the CDOM analysis, 300 mL of seawater was collected from 2–5 m depth using a Niskin bottle and stored in amber bottles in darkness at 4°C until further processing. Within a few hours of collection, the water samples were filtered through a 0.22 *μ*m polycarbonate filter prerinsed with Milli‐Q water. Using a peristaltic pump (flow rate ~ 1 mL min^−1^), the samples were injected into a 2.5 m liquid waveguide capillary cell (LWCC) 61 coupled with a miniature fiber‐optic spectrometer (USB2000 + , preconfigured to 200–850 nm; Ocean Optics Inc.) and a dual lamp (tungsten and deuterium) light source (DH‐2000; Ocean Optics Inc.). Absorbance was determined for the wavelength range 250–750 nm at a resolution of ~0.4 nm. The sample spectrum was compared against the spectrum of Milli‐Q water after the refractive index differences were corrected by subtracting the mean value of OD_CDOM_ (*λ*) between 683 and 687 nm (OD_null_,_CDOM_) from the whole spectrum, following Babin *et al*. 62. The CDOM absorption coefficients (*a*_CDOM_ (*λ*), m^−1^) were calculated using Eqn (1),

(1) $$a_{\mathrm{CDOM}}\left(\lambda \right)=2.303\frac{\left[{\text{OD}}_{\mathrm{CDOM}}\left(\lambda \right)-{\text{OD}}_{\mathrm{null},\mathrm{CDOM}}\right]}{l}$$

where *l* is the optical path length (m) and 2.303 is the factor used to convert base *e* to base 10 logarithms. We report *a*_CDOM_ for the individual wavelengths in the range 270–600 nm. Furthermore, we have determined the slopes in the regions 275–295 nm (*S*_275–295_) and 350–400 nm (*S*_350–400_), and the ratio of these slopes (*S*_R_ = *S*_275–295_ : *S*_350–400_). Spectral slopes reported were calculated using linear regression of the log‐transformed *a*_CDOM_ spectra, following the method of Helms *et al*. 63.

### Downwelling diffuse attenuation coefficient (K_d_)

Initially, a visual quality check was performed on the spectroradiometer profile data to remove any erroneous irradiance values, such as obvious outliers. Additionally, for each spectral measurement at every depth, we approximated the instantaneous downwelling irradiance for the integrated UV‐B (280–320 nm) and UV‐A (320–400 nm) using trapezoidal integration. For the UV‐B spectrum, the integration was performed using the instantaneous downwelling irradiance (*E*_d_) of the 305, 313 and 320 nm channels, while for the UV‐A spectrum, we used the *E*_d_ of the 320, 340, 380 and 395 nm channels. The instrument detection limit for the most attenuated wavelength (i.e., 305 nm) was generally reached at a depth of 30–35 meters, so we calculated the spectral diffuse attenuation coefficient (*K*_d_ (*λ*)) for all UV channels (305, 313, 320, 340, 380 and 395 nm) down to 30 m depth and, for PAR, down to the deepest sampling depth of approximately 40 m. Irradiance *versus* depth profiles from the spectroradiometer casts were used to calculate *K*_d_ (*λ*) by performing a linear regression of the natural logarithm using Lambert–Beer's law,

(2) $$E_{d}\left(Z,\lambda \right)=E_{d}\left(-0,\lambda \right)e^{-K_{d}\left(\lambda \right)Z}$$

where *E*_d_ (Z, *λ*) is the downwelling irradiance at depth *Z* and *E*_d_ (–0, *λ*) is the irradiance just beneath the air–water interface. The slope of the linearegression informed about the value of *K*_d_ (*λ*). Generally, the fitting process was highly robust with correlation coefficients of R^2^ > 0.99. Since *Z* (depth) was measured in meters, the unit of *K*_d_ (*λ*) is m^−1^.

### Percent attenuation depth (*Z*_%_) *and mixed layer depth (MLD)*

For each UV wavelength (305, 313, 320, 340, 380 and 395 nm), as well as the integrated PAR spectrum (400–700 nm), we calculated the 10% and 1% attenuation depths (*Z*_*n*%_), which are defined as the depths at which irradiance is reduced to *n*% of the value immediately below the water surface. The depths were calculated using the fraction of surface irradiance (*E*_d_ (Z, *λ*)/*E*_d_ (–0, *λ*)) and *K*_d_, as shown in Eqn (3).

(3) $$Z_{n\%}=\frac{\ln{\left[\frac{E_{d}\left(Z,\lambda \right)}{E_{d}\left(-0,\lambda \right)}\right]}^{-1}}{K_{d}}$$

The 10% and 1% attenuation depths are especially significant in terms of the PAR spectrum, as they are traditionally used to identify the midpoint and bottom of the euphotic zone, respectively 3, 64. In the context of UV, Kuwahara *et al*. 43 defined the 10% penetration depth as the biologically effective optical depth and the 1% penetration depth as the implicit UV within the euphotic zone.

Additionally, the mixed layer depth (MLD), an index of the stability of the water column, was calculated from the CTD profiles as the shallowest depth at which the water density (*σ*_*θ*_) differs from surface values by more than 0.05 kg m^−3^ 65. For stations where salinity data could not be recorded due to an instrument malfunction, we calculated the MLD using a threshold value in temperature (Δ*T* = 0.2°C), as described by de Boyer Montegut *et al*. 66.

### Statistical analysis

Linear regression analyses were performed to explore the linear relationship between *K*_d_ (*λ*) and the two independent variables *a*_CDOM_ (*λ*) and Chl‐*a* concentration. Data analyses and visualizations were performed using the software packages JMP Pro 13 (SAS Institute Inc., Cary, USA) and GraphPad Prism 7 (GraphPad Software, La Jolla, USA).

## Results

### Attenuation coefficient (*K*_d_)

**Figure 1:**
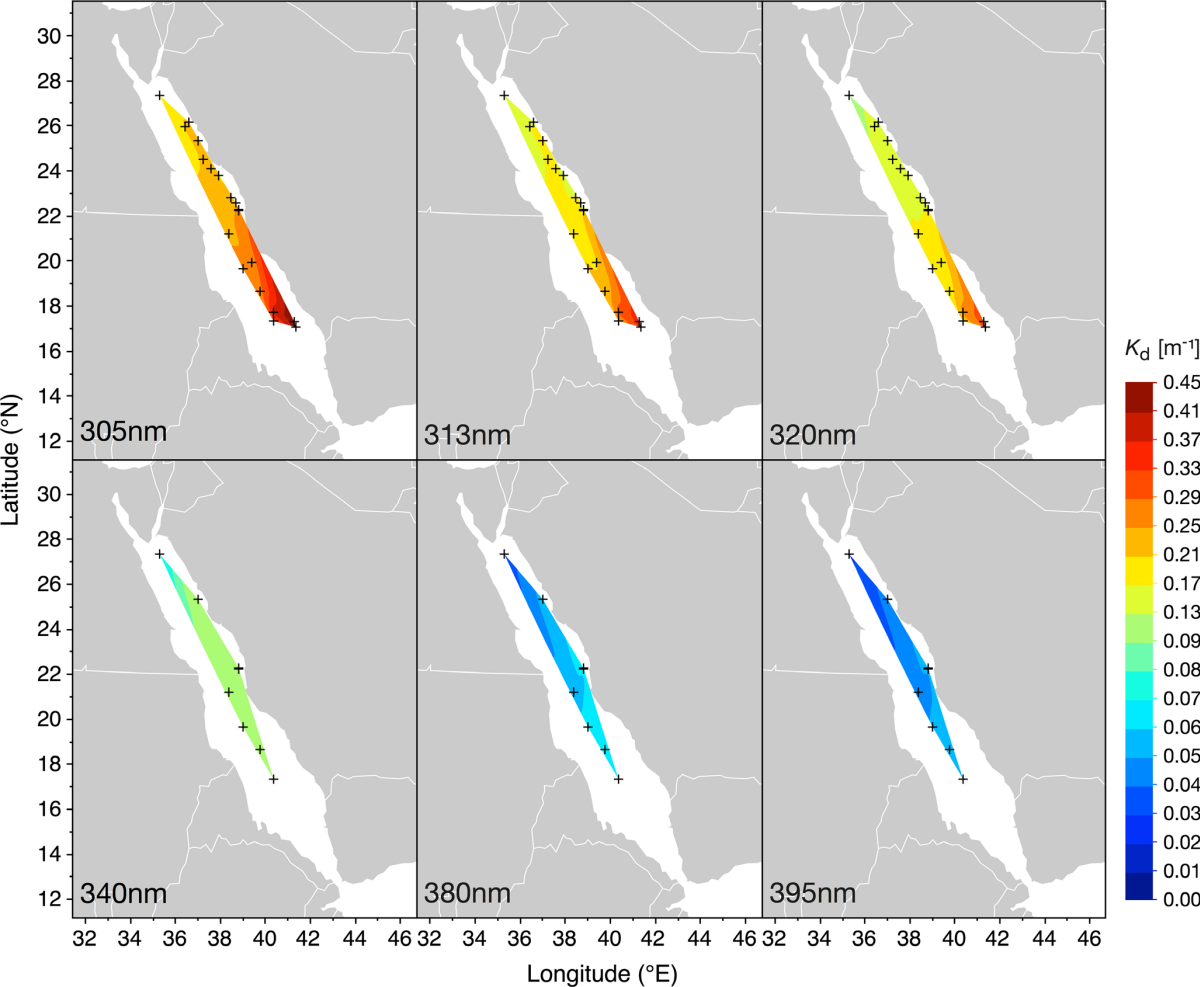
Downwelling diffuse attenuation coefficients (*K*_d_) for three UV‐B wavelengths (305, 313 and 320 nm; top panels) and three UV‐A wavelengths (340, 380 and 395 nm; bottom panels), measured during four Red Sea cruises between October 2016 and March 2018. Contours were smoothed at a level of 0.05.

UV attenuation varied greatly among the stations and wavelengths. For the UV‐B wavelengths 305, 313 and 320 nm, *K*_d_ ranged from 0.168 to 0.451 m^−1^, 0.130 to 0.357 m^−1^ and 0.110 to 0.320 m^−1^, respectively (Fig. 1, top panels). For the three UV‐A wavelengths, *K*_d_ varied with the ranges 0.070–0.143 m^−1^ (340 nm), 0.034–0.073 m^−1^ (380 nm) and 0.028–0.062 m^−1^ (395 nm) (Fig. 1, bottom panels).

**Figure 2:**
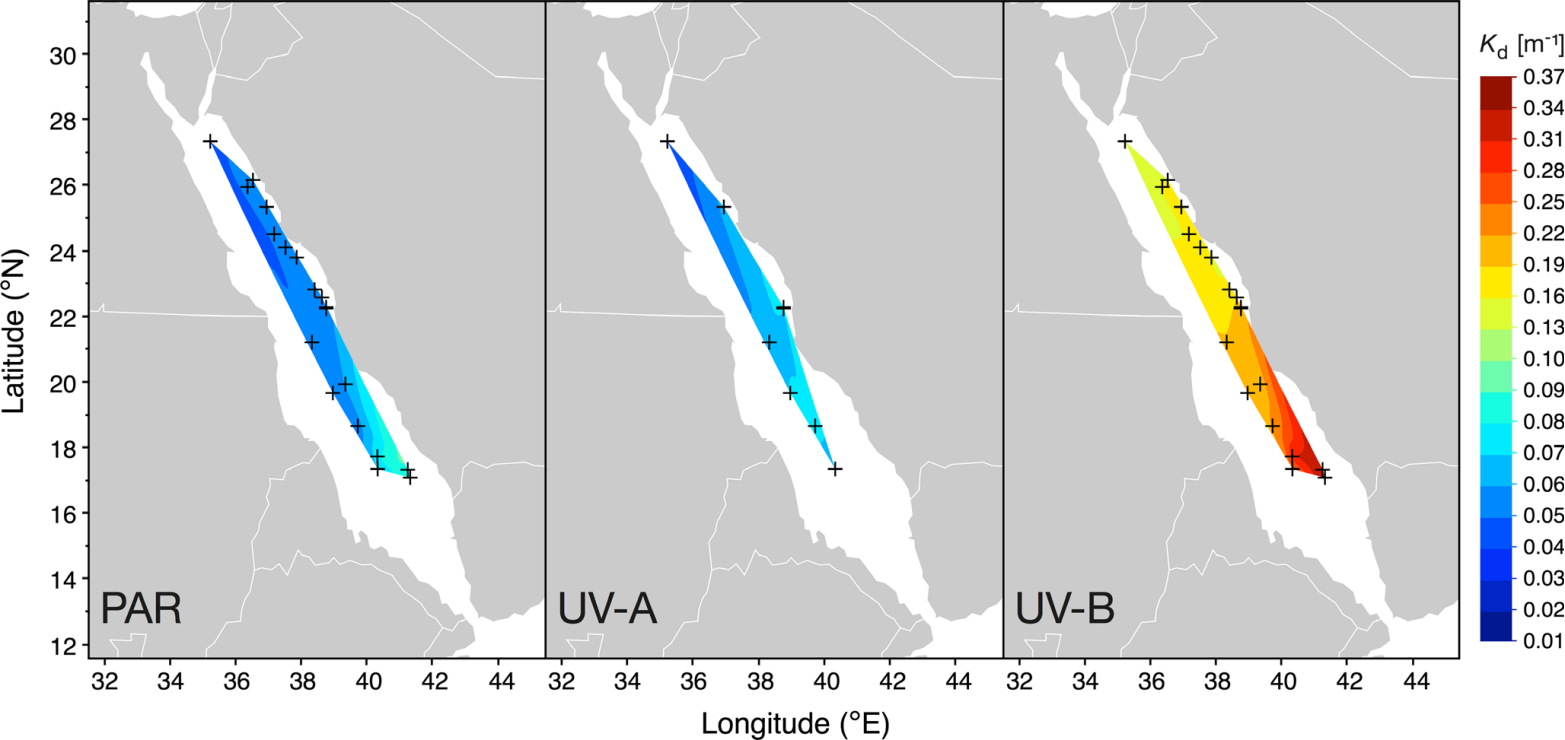
Downwelling diffuse attenuation coefficients (*K*_d_) for the integrated PAR (400–700 nm) UV‐A (320–400 nm) and UV‐B spectra (280–320 nm). PAR and UV‐B were measured during four Red Sea cruises between October 2016 and March 2018, and UV‐A was only measured as part of two cruises in August 2017 and March 2018. Contours were smoothed at a level of 0.05.

We identified the highest transparency to UV‐A and UV‐B in August 2017 at our northernmost sampling station, off the coast of Duba (27.3°N, 34.8°E) and approximately 75 km south of the entrance to the Gulf of Aqaba. The strongest UV attenuation was measured in November 2016 at three stations in the far south of the Red Sea, close to the Farasan Islands (~17°N, 41°E) (Fig. 2, middle and right panel). Regarding *K*_d_ of the UV‐A and UV‐B spectra as a whole, the spatial pattern was the same as for individual UV wavelengths. Attenuation was highest around the Farasan Islands (maximum *K*_d_ (UV‐B): 0.354 m^−1^; maximum *K*_d_ (UV‐A): 0.083 m^−1^), while the waters in the far north of the Red Sea were the most transparent (minimum *K*_d_ (UV‐B): 0.131 m^−1^; minimum *K*_d_ (UV‐A): 0.041 m^−1^) (Fig. 2, middle and right panel).

For PAR, we identified a slightly different pattern of attenuation. We found the least transparent waters (*K*_d_ (PAR): 0.096 m^−1^) approximately 50 km north of the Farasan Islands (17.3°N, 41.4°E) in November 2016, whereas the most transparent waters (*K*_d_ (PAR): 0.039 m^−1^) were sampled in the central Red Sea (21.2°N, 38.3°E) in August 2017 (Fig. 2, left panel). When the same station in the central Red Sea was sampled again in March 2018, we found a *K*_d_ (PAR) of 0.060 m^−1^.

In autumn 2016, we identified a pronounced north‐to‐south gradient in the UV attenuation, where the lowest attenuation (*K*_d_ (UV‐B): 0.143 m^−1^; *K*_d_ (UV‐A): not determined) was measured at the northernmost station off the coast of Al Wajh (26.1°N, 36.3°E) (Fig. 2, right panel). In summer 2017, five stations were sampled between Duba and the Farasan Banks (17.4°N, 40.4°E). During that time, the UV attenuation was strongest at the two coastal stations (*K*_d_ (UV‐B): 0.199–0.220 m^−1^; *K*_d_ (UV‐A): 0.063–0.076 m^−1^), whereas the three offshore stations located at the mid‐ridge were all highly transparent to UV (*K*_d_ (UV‐B): 0.131–0.178 m^−1^; *K*_d_ (UV‐A): 0.041–0.059 m^−1^). In early spring 2018, a further five stations were sampled. The UV‐B attenuation varied only marginally across those five stations (*K*_d_ (UV‐B): 0.210–0.246 m^−1^; *K*_d_ (UV‐A): 0.068–0.083 m^−1^), with no distinct north‐to‐south or onshore‐offshore gradient in water transparency. Across all cruises, however, we identified that the *K*_d_ of UV‐B wavelengths and PAR showed a negative and strongly significant linear relationship with latitude (*P* < 0.001).

### Percent attenuation depth (*Z*_%_)

The depths to which 10% and 1% of the surface irradiance reached (i.e., *Z*_10%_ and *Z*_1%_) varied considerably depending on the wavelength, with the shortest UV wavelengths reaching the shallowest depths. For example, the average *Z*_10%_ values for the three UV‐B wavelengths 305, 313 and 320 nm were 9.8, 12.1 and 13.9 m, respectively; for the UV‐A wavelengths, the average *Z*_10%_ values were 22.0 m (340 nm), 43.3 m (380 nm) and 52.4 m (395 nm) (Table 2). The maximum *Z*_10%_ was 17.6 m for the UV‐B spectrum and 56.6 m for the UV‐A spectrum. Interestingly, the maximum *Z*_10%_ of UV (measured for 395 nm) was 82.7 m, which was deeper than the maximum *Z*_10%_ of PAR (58.4 m). We also found that the maximum *Z*_1%_ of 395 nm (165.5 m) was deeper than the deepest recorded *Z*_1%_ of PAR (116.8 m) (Table 2). The largest *Z*_1%_ values measured for the other wavelengths were 135.3 m (380 nm), 66.2 m (340 nm), 41.9 m (320 nm), 35.3 m (313 nm) and 27.3 m (305 nm). For the integrated UV‐B and UV‐A spectra, the maximum *Z*_1%_ values were 35.2 and 112.7 m, respectively.

**Table 2:** Percent attenuation depths (*Z*_10%_ and *Z*_1%_; in m) for both individual UV wavelengths and the integrated UV‐B (280–320 nm), UV‐A (320–400 nm) and PAR (400–700 nm) spectra. For locations of sampling stations, see Table 1

Date	*Z*_10%_ (m)									*Z*_1%_ (m)								
	305 nm	313 nm	320 nm	340 nm	380 nm	395 nm	UV‐B	UV‐A	PAR	305 nm	313 nm	320 nm	340 nm	380 nm	395 nm	UV‐B	UV‐A	PAR
03.10.2016	10.8	16.4	17.4	N/A	N/A	N/A	15.6	N/A	51.5	21.7	32.7	34.7	N/A	N/A	N/A	31.1	N/A	102.9
04.10.2016	10.8	13.3	14.9	N/A	N/A	N/A	14.5	N/A	45.6	21.6	26.5	29.8	N/A	N/A	N/A	29.0	N/A	91.3
06.10.2016	11.3	14.4	16.6	N/A	N/A	N/A	16.1	N/A	46.4	22.6	28.7	33.2	N/A	N/A	N/A	32.2	N/A	92.7
07.10.2016	11.5	15.3	17.9	N/A	N/A	N/A	15.4	N/A	36.3	23.0	30.6	35.8	N/A	N/A	N/A	30.9	N/A	72.6
08.10.2016	9.9	12.6	14.5	N/A	N/A	N/A	13.0	N/A	43.1	19.8	25.2	29.0	N/A	N/A	N/A	25.9	N/A	86.1
22.11.2016	8.9	11.6	13.3	N/A	N/A	N/A	11.8	N/A	38.6	17.9	23.2	26.6	N/A	N/A	N/A	23.5	N/A	77.2
23.11.2016	5.3	6.8	7.7	N/A	N/A	N/A	6.8	N/A	27.1	10.5	13.5	15.4	N/A	N/A	N/A	13.7	N/A	54.2
24.11.2016	5.2	6.7	7.7	N/A	N/A	N/A	6.7	N/A	31.2	10.4	13.5	15.4	N/A	N/A	N/A	13.4	N/A	62.4
25.11.2016	5.1	6.4	7.2	N/A	N/A	N/A	6.5	N/A	23.9	10.2	12.9	14.4	N/A	N/A	N/A	13.0	N/A	47.7
29.11.2016	10.1	12.8	14.9	N/A	N/A	N/A	12.9	N/A	41.1	20.1	25.6	29.8	N/A	N/A	N/A	25.9	N/A	82.3
30.11.2016	9.0	12.1	14.0	N/A	N/A	N/A	11.9	N/A	42.4	17.9	24.2	27.9	N/A	N/A	N/A	23.8	N/A	84.9
01.12.2016	9.9	13.2	15.3	N/A	N/A	N/A	13.4	N/A	41.7	19.8	26.5	30.6	N/A	N/A	N/A	26.8	N/A	83.5
02.08.2017	8.5	9.6	11.8	18.0	35.2	42.7	9.3	27.7	39.1	17.1	19.2	23.5	36.0	70.4	85.3	18.7	55.4	78.2
05.08.2017	13.7	17.7	20.9	33.1	67.6	82.1	10.6	32.4	48.1	27.3	35.3	41.9	66.2	135.3	164.3	21.2	64.9	96.2
07.08.2017	10.2	12.3	14.5	21.6	42.2	50.7	10.4	32.5	40.4	20.4	24.7	29.0	43.3	84.4	101.3	20.7	65.1	80.7
14.08.2017	10.2	12.8	15.1	23.7	45.5	54.0	10.6	29.0	42.6	20.5	25.6	30.2	47.5	90.9	108.0	21.3	58.0	85.2
16.08.2017	13.1	16.7	16.7	32.3	67.4	82.7	11.0	33.9	58.4	26.3	33.5	33.5	64.5	134.9	165.5	21.9	67.8	116.8
16.03.2018	7.6	9.2	10.5	16.1	31.4	38.1	10.5	30.5	38.5	15.1	18.3	21.0	32.1	62.8	76.2	21.0	60.9	76.9
17.03.2018	8.5	10.5	12.3	18.8	36.1	44.9	17.6	56.4	39.9	17.0	21.1	24.5	37.6	72.2	89.8	35.2	112.7	79.9
18.03.2018	8.3	10.3	12.0	18.5	36.7	44.8	11.6	36.3	43.4	16.6	20.6	24.0	36.9	73.4	89.7	23.2	72.7	86.8
19.03.2018	8.6	10.5	12.2	18.1	32.4	37.3	12.9	39.3	32.2	17.2	21.1	24.4	36.3	64.8	74.6	25.9	78.6	64.3
21.03.2018	8.8	10.9	12.7	19.6	38.9	47.1	16.9	55.7	45.1	17.5	21.8	25.4	39.1	77.7	94.3	33.9	111.4	90.2

### CDOM absorption coefficient (*a*_CDOM_)

**Figure 3:**
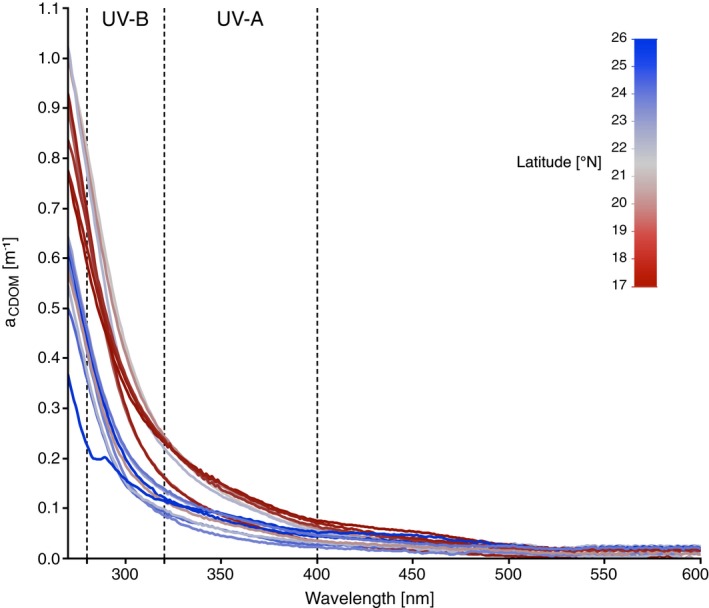
CDOM absorbance (*a*_CDOM_ in m^−1^) spectra (displayed for 270–600 nm) of water samples collected in the Red Sea from 5 m depth during three cruises between October 2016 and March 2018. Color of the spectrum line indicates the latitude of the sampling station.

UV absorption by CDOM varied markedly across the Red Sea and decreased with increasing wavelength. For 305 nm, we measured a minimum and maximum CDOM absorption coefficient (*a*_CDOM_ (305 nm)) of 0.128 and 0.324 m^−1^ (mean: 0.207 m^−1^), respectively. For 313 and 320 nm, *a*_CDOM_ varied from 0.104 to 0.272 m^−1^ (mean: 0.172 m^−1^) and from 0.086 to 0.239 m^−1^ (mean: 0.150 m^−1^), respectively (Fig. 3). For the wavelengths of the UV‐A spectrum (340, 380 and 395 nm), the average *a*_CDOM_ was lower and with a narrower range than for the UV‐B spectrum. Specifically, *a*_CDOM_ (340 nm) varied between 0.055 and 0.171 m^−1^ (mean: 0.108 m^−1^) and *a*_CDOM_ (380 nm) from 0.028 to 0.097 m^−1^ (mean: 0.060 m^−1^); on average, 395 nm was absorbed the least by CDOM (mean *a*_CDOM_ (395 nm): 0.049 m^−1^) with a minimum and maximum *a*_CDOM_ (395 nm) of 0.023 and 0.079 m^−1^, respectively (Fig. 3).

**Figure 4:**
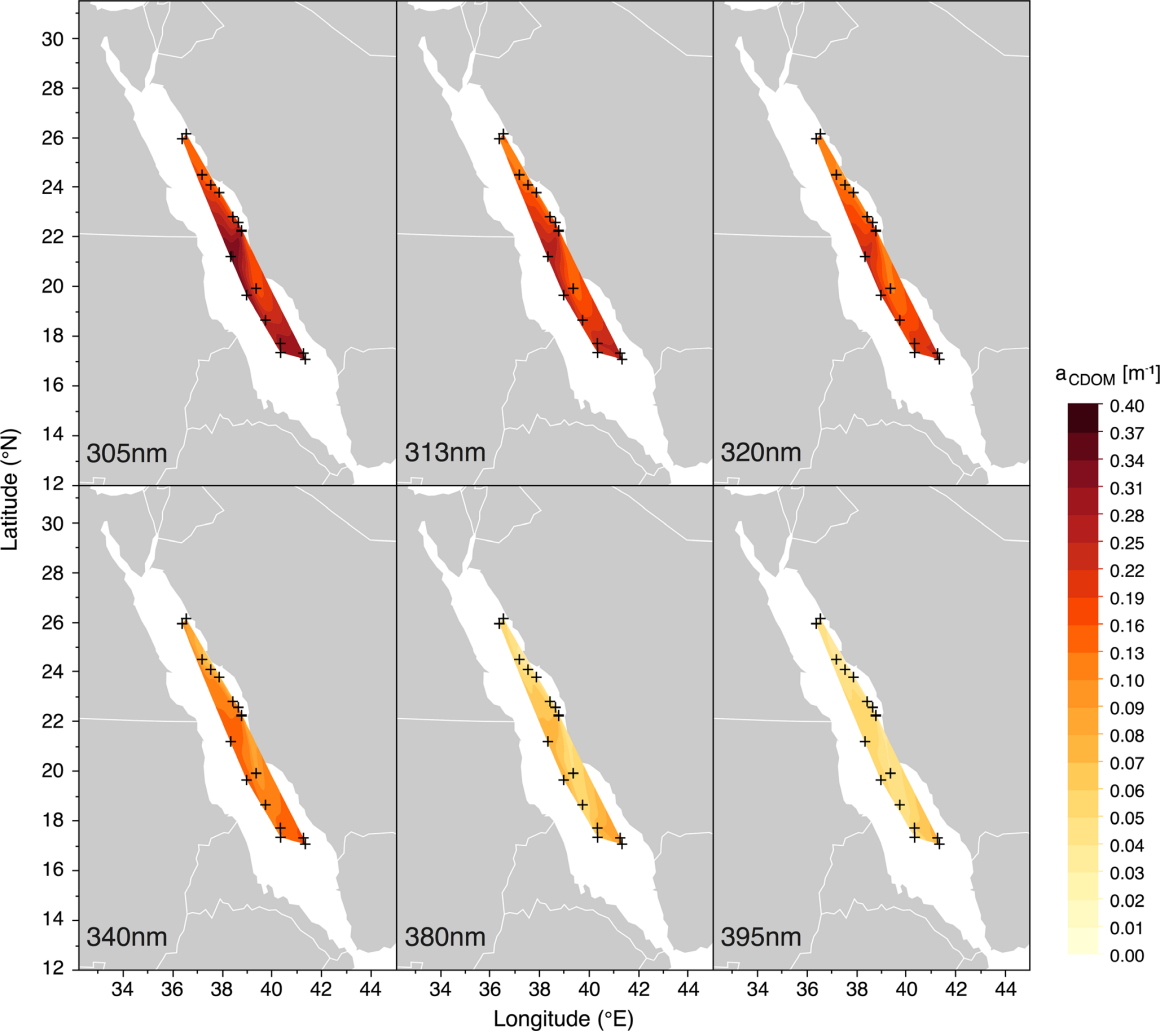
Spatial pattern of CDOM absorbance (*a*_CDOM_ in m^−1^) for three UV‐B wavelengths (305, 313 and 320 nm; top panels) and three UV‐A wavelengths (340, 380 and 395 nm; bottom panels), measured during three Red Sea cruises between October 2016 and March 2018. Crosses represent the location of each sampling point, and contours were smoothed at a level of 0.05.

Across all the UV wavelengths, the highest *a*_CDOM_ were measured close to the Farasan Islands (17°N) in late November 2016. The lowest *a*_CDOM_, on the other hand, were measured in early October 2016 off the coast of Yanbu (24°N) (Fig. 4).

The spectral slopes ranged from 0.019 to 0.046 nm^−1^ (*S*_275–295_) and from 0.011 to 0.019 nm^−1^ (*S*_350–400_), while the values of S_R_ varied from 1.69 to 4.09 (Table 1). No obvious spatial pattern could be observed regarding the slopes and ratio; however, those parameters exhibited a distinct temporal pattern. Specifically, *S*_275–295_ was highest in October (0.044 nm^−1^ ± 0.03) while for *S*_350–400,_ the highest values could be observed in March (0.018 nm^−1^ ± 0.001). The ratio S_R_ on the other hand seemed to gradually decrease from fall to winter, with mean values of 3.33 ± 0.57 (Oct), 2.28 ± 0.55 (Nov) and 1.96 ± 0.21 (Mar).

### Chlorophyll‐*a*

**Figure 5:**
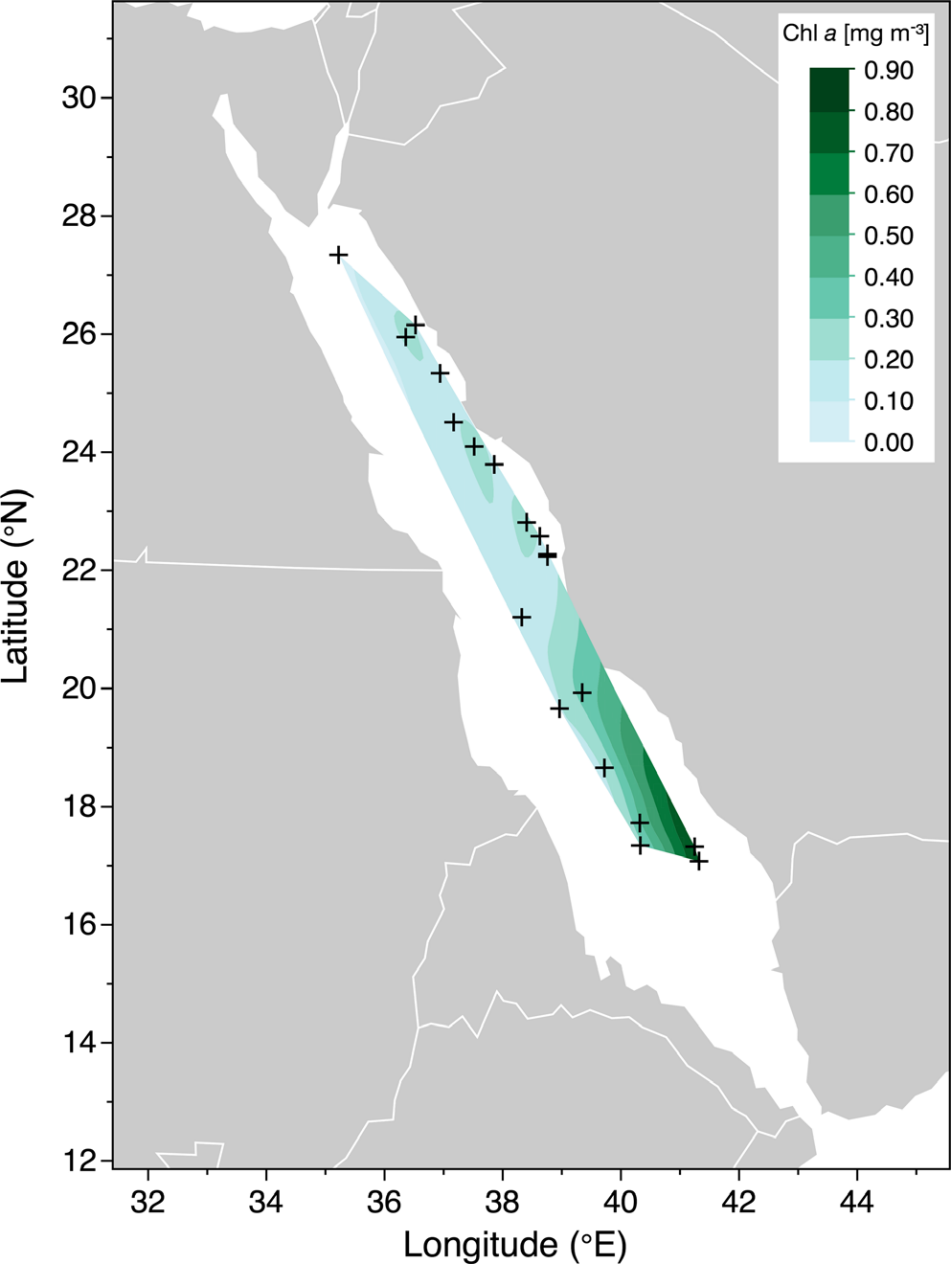
Concentration of Chl‐*a* (mg m^−3^) in Red Sea surface waters (5 m depth) determined during three cruises between October 2016 and March 2018. Crosses mark the location of each sampling station. Contour lines were smoothed at a level of 0.05.

The amount of Chl‐*a* in the surface waters varied between 0.07 and 0.83 mg m^−3^, with the lowest and highest concentrations measured in the far northern Red Sea (27.3°N, 34.8°E) in August 2017 and at a sampling site close to the Farasan Islands (17.3°N, 41.4°E) in November 2016, respectively (Table 1). Surface Chl‐*a* generally increased in concentration from south to north, exhibiting a latitudinal gradient (Fig. 5). Additionally, we could observe a clear seasonal pattern with the highest Chl‐*a* concentrations being present in fall when the surface waters were deeply mixed (max. MLD: 72 m) (Table 1). In contrast, minimal Chl‐*a* levels were measured in summer when the upper layer of the water column was highly stratified and the MLD was found to be as shallow as 11 m.

### Attenuators of UV

**Figure 6:**
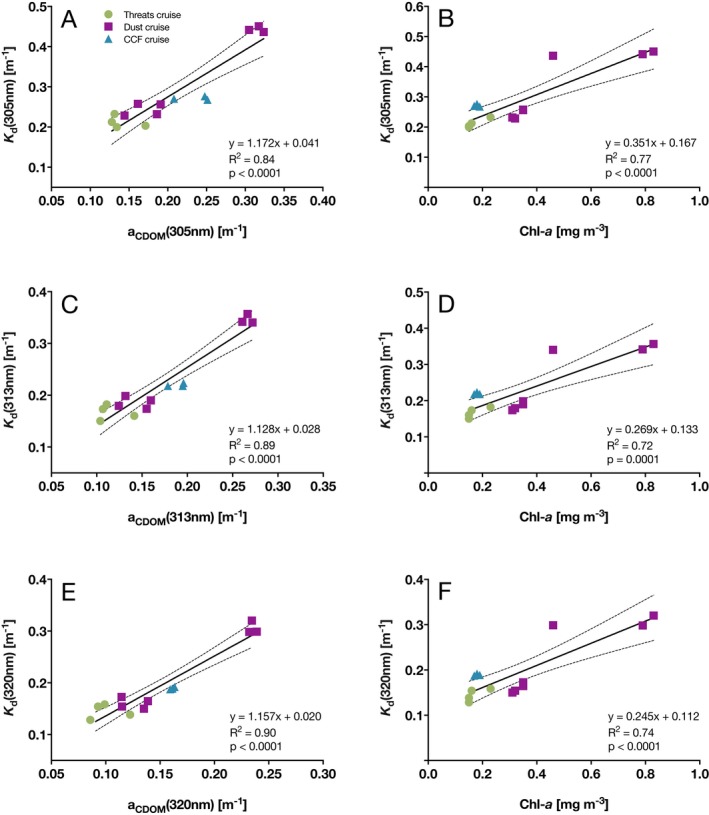
Analysis of the relationship between the downwelling attenuation coefficient (*K*_d_ (*λ*)) and the two parameters *a*_CDOM_ (*λ*) (left panels) and Chl‐*a* concentration (right panels) for the wavelengths 305 nm (A, B), 313 nm (C, D) and 320 nm (E, F). Data are from the *Threats* cruise (October 2016, green circles), *Dust* cruise (November–December 2016, purple squares) and *CCF* cruise (March 2018, blue triangles). The solid line represents the regression line; dashed lines indicate 95% CIs.

Based on data from three cruises (*n* = 14), we identified a positive linear relationship between *K*_d_ (*λ*) and the two parameters Chl‐*a* concentration and *a*_CDOM_ (Fig. 6). For each of the three UV‐B wavelengths (305, 313 and 320 nm), we found that the slope of the regression line for *a*_CDOM_ was greater than that of the Chl‐*a* concentration. Furthermore, the slope of *a*_CDOM_ (1.172, 1.128 and 1.157 for 305, 313 and 320 nm, respectively) increased with increasing wavelength, whereas the opposite pattern was identified for the slope of Chl‐*a* (0.351, 0.269 and 0.245 for 305, 313 and 320 nm, respectively).

For each of the UV‐B wavelengths, the coefficient of determination (*R* ^2^) was higher between *a*_CDOM_ and *K*_d_ (*λ*) than between the Chl‐*a* concentration and *K*_d_ (*λ*) (Fig. 6). The strongest correlation (*R* ^2^ = 0.90, y = 1.157*x* + 0.020, *P* < 0.0001) was found between *a*_CDOM_ (320 nm) and *K*_d_ (320 nm), whereas the weakest correlation was identified between the concentration of Chl‐*a* and *K*_d_ (305 nm) (*R* ^2^ = 0.77, *y* = 0.351*x* + 0.167, *P* < 0.0001).

## Discussion

Our findings evidence that the degree to which ultraviolet radiation (UV) is attenuated in the Red Sea water column varies considerably across latitudes. The far northern Red Sea is the most optically transparent to UV, whereas the waters around the Farasan Islands in the south of the Red Sea basin strongly attenuate UV. Our results further indicate that the Red Sea might be more optically transparent to both UV and PAR than previously described. For example, Dishon *et al*. 49 calculated a minimum *K*_d_ for the integrated UV‐B spectrum (300–320 nm) of ~0.159 m^−1^ in the Gulf of Aqaba, which is located in the far north of the Red Sea. In comparison, we obtained an even lower value of 0.131 m^−1^ for the UV‐B spectrum (280–320 nm) in the northern Red Sea off the coast of Duba (Fig. 2). Additionally, we identified a minimum attenuation coefficient for PAR (*K*_d_ (PAR)) of 0.039 m^−1^, which is lower than the *K*_d_ (PAR) values reported for the Red Sea by both Dishon *et al*. 49 (*K*_d_ (PAR): 0.056 m^−1^) and Stambler 8 (*K*_d_ (PAR): 0.049 m^−1^). Moreover, our results show that, during summer in the northern Red Sea, UV‐A (*Z*_10%_ (340 nm): 33 m) and UV‐B (*Z*_10%_ (305 nm): 14 m) wavelengths can penetrate into the water column as deep as, or even deeper than, in the highly transparent western Mediterranean Sea (*Z*_10%_ (340 nm): 15–21 m; *Z*_10%_ (305 nm): 10–11 m) 67, 68 or open ocean waters, such as the central subtropical Atlantic Ocean (*Z*_10%_ (340 nm): 35 m; *Z*_10%_ (305 nm): 16 m) or the Gulf of Mexico (*Z*_10%_ (340 nm): 37 m; *Z*_10%_ (305 nm): 13 m) 69, 70, 71.

The observed latitudinal pattern of UV attenuation is governed by the Red Sea's natural gradient in physical and biogeochemical properties. The northern Red Sea and the Gulf of Aqaba are both characterized by ultra‐oligotrophic waters, with minimal concentrations of Chl‐*a* and CDOM for the majority of the year 6, 39, 72, 73. However, the Gulf of Aqaba becomes deeply mixed in spring, favoring phytoplankton growth, whereas the northern Red Sea is stratified during that time. This difference was reflected in our results by the attenuation coefficients we measured for UV‐A, UV‐B and PAR in the northern Red Sea, which were lower than those previously described for the Gulf of Aqaba 8, 49. In the central and southern Red Sea, we found that UV attenuation gradually increased toward the south, where water turbidity and nutrient concentrations were highest, and Chl‐*a* reached concentrations of 1–3 mg Chl‐*a* m^−3^ 5, 6, 12, 74 due to the influx of nutrient‐rich water from the Gulf of Aden through the strait of Bab‐el‐Mandeb 74, 75. While the southernmost area of the Red Sea was not sampled as part of the present study, we measured the strongest UV attenuation at our three southernmost sampling stations close to the Farasan Banks.

The main drivers determining UV attenuation are UV‐absorbing and UV‐scattering substances present in the water column. Of these, Chl‐*a,* which is indicative of phytoplanktonic biomass, and CDOM are the most significant 3. Both of those water constituents exhibited a distinct seasonality in the Red Sea during our study. Furthermore, we measured the lowest attenuation for all UV wavelengths off Duba during summer when the water column was highly stratified (MLD: 22 m) and the concentration of Chl‐*a* and the absorption by CDOM were lowest across the Red Sea basin. In contrast, the strongest UV attenuation was observed close to the Farasan Banks during winter and spring, coinciding with higher Chl‐*a* and CDOM concentrations in the water column and intensified vertical mixing processes (MLD: 51 m).

We found that *K*_d_ for the UV‐B wavelengths (305, 313 and 320 nm) was significantly (*P* < 0.05) correlated with the Chl‐*a* concentration and *a*_CDOM_. For example, *K*_d_ increased more per unit of Chl‐*a* for shorter UV wavelengths, based on the slope coefficients for 320 (*y* = 0.25*x*), 313 nm (*y* = 0.27*x*) and 305 nm (*y* = 0.35*x*). The coefficients of determination (*R* ^2^) between *K*_d_ and the Chl‐*a* concentration were generally high, but the highest *R* ^2^ was identified for 305 nm (*R* ^2^ = 0.77), confirming that Chl‐*a* plays a larger role in the attenuation of short UV‐B wavelengths (e.g. 305 nm) than those closer to the UV‐A spectrum (i.e. 313 nm and 320 nm). In comparison, a study of the South Pacific Ocean and the Mediterranean Sea reported an average correlation coefficient between chlorophyll concentration and *K*_d_ (320 nm) of *R* ^2^ = 0.694 37. However, Dishon *et al*. 49 found an even stronger correlation (*R* ^2^ = 0.78) between the Chl‐*a* concentration and light attenuation (i.e. *K*_d_ (PAR)), which suggests that Chl‐*a* predominantly contributes to the absorption of radiation in the PAR spectrum. In contrast, a study by Stambler 8 identified that Red Sea phytoplankton strongly absorb in the UV spectrum and that the absorption coefficients for UV wavelengths exceed the coefficient for 675 nm (the major absorption peak of Chl‐*a* in the PAR spectrum). However, we note that Chl‐*a* concentration is merely used as a proxy for phytoplankton biomass and that other UV‐absorbing or UV‐screening cell components besides Chl‐*a* could be responsible for the strong UV attenuation by phytoplankton, such as mycosporine‐like amino acids (MAAs) or scytonemin 76, 77, 78.

Regarding the overall contribution of phytoplankton and CDOM toward UV attenuation, we identified CDOM as the key modulator. Using the data displayed in Fig. 6, we calculated that *a*_CDOM_ (*λ*) contributed an estimated 73 ± 11.1%, 77 ± 10.2% and 77 ± 10.0% toward the downwelling *K*_d_ values of the 305, 313 and 320 nm wavelengths, respectively. This finding is in agreement with other studies on natural marine waters that identified CDOM as the principal attenuator of UV 33, 69, 79, whereas the contribution of phytoplankton (<20%) and nonpigmented particles (<15%) toward the total absorption of UV wavelengths was reported to be comparatively low 21. Additionally, our values fall within the range reported for the southeastern Pacific Ocean by Tedetti *et al*. 80, who found that the relative contribution of CDOM to the total absorption of 305 nm in surface waters varied between 71% and 95% (compared to 73% in our study). In the present study, we only analyzed CDOM samples collected from 5 m depth, yet, it is known that the concentration and absorption of CDOM can vary considerably along the water column 31, including in the Red Sea 39, due to changes in the source material, microbial activity and photobleaching that often result in higher CDOM concentrations found below the upper mixed layer 22, 81. A large proportion of our spectroradiometer profiles did not reach below the mixed layer, while for those that went beneath it, we did not notice an obvious change in *K*_d_ at the boundary between the two layers, suggesting no marked change in the concentration of CDOM from 5 m to ~40 m (our maximum sampling depth), possibly due to the absence of high amounts of source material. Large quantities of CDOM are usually transported from land into coastal waters by rivers 23, 26, 27, 82, however, the Red Sea lacks riverine inputs 83, 84, contributing toward its high transparency. Our finding that *a*_CDOM_ and UV attenuation were strongest at the stations around the Farasan Islands could be due to CDOM sources from coastal marine vegetation, such as mangroves and seagrasses 29, 85, 86, which are abundant in this area characterized by islands, shallow sandbanks and reefs 60, 74, 87.

Previous studies suggested that both the northern Red Sea and the Gulf of Aqaba are Case‐1 waters 8, 88; that is, phytoplankton and their associated CDOM and detritus degradation products govern the optical properties 89, 90. Our findings indicate that not just the northern Red Sea but rather the vast majority of the basin can be classified as Case‐1 waters, since CDOM closely mirrors the optical properties of phytoplankton (see Figs 4 and 5) and the values of *S*_275–295_ were larger than *S*_350–400_ (i.e. *S*_R_ > 1) (Table 1), which is typical for low molecular weight (LMW) CDOM in open ocean waters 63, 91. This finding also suggests that the bulk of CDOM in the Red Sea is heavily photobleached and of marine rather than terrigenous origin 63, 89, 92. In fact, this closely coupled relationship between Chl‐*a* and CDOM in the Red Sea, confirming the predominantly phytoplanktonic origin of CDOM, was recently described by Kheireddine *et al*. 39. One exception to the Case‐1 classification could be the Farasan Islands in the far south of the Red Sea, an area previously categorized as Case‐2 waters based on remote sensing data 93. At the Farasan Banks, however, we did not find an unusually low S_R_ in this region to support this hypothesis. Therefore, a thorough study should be carried out to determine whether phytoplankton and its associated degradation products or benthic marine vegetation represent the primary source of CDOM and, therefore, the main driver of the high UV attenuation observed in this area.

## Conclusion and Future Outlook

Here, we confirm that the Red Sea is indeed highly transparent to both UV‐A (maximum *Z*_10%_ (UV‐A): 57 m) and UV‐B (maximum *Z*_10%_ (UV‐B): 18 m) and that its bio‐optical properties are comparable to those of the clearest ocean waters. The high transparency of the Red Sea, in combination with the intense incident UV and minimal cloud cover 5 in this region, means that the biota in its surface waters is exposed to extreme doses of damaging UV. Therefore, UV is likely a key ecological factor governing the distribution, metabolism and survival of organisms in the upper euphotic zone of the Red Sea, as previously proposed for a wide variety of Red Sea taxa, including zooplankton 94, soft corals 95 and picophytoplankton 50, 96. While the northern Red Sea was found to be more transparent to wavelengths of the UV spectrum than its southern counterpart, its higher latitude means that the incident solar radiation is less intense. Consequently, the maximum UV doses in the water column of the northern and southern Red Sea might be comparable in magnitude.

Ongoing and complex atmospheric and environmental changes 97 impose a high degree of uncertainty onto future projections regarding the intensity of incident UV and UV attenuation by Red Sea waters. For instance, current models predict that the total amount of stratospheric ozone over the tropics might decrease by the end of this century due to the emission of greenhouse gases and changes in the global circulation pattern of ozone 98, 99. Similarly, while upper stratospheric ozone seems to show renewed growth, a recent study by Ball *et al*. 100 found that lower stratospheric ozone levels are being depleted, which may be linked to the persistent emission of ozone‐reducing chlorofluorohydrocarbons (CFCs) despite the Montreal Protocol banning their use 101, 102. Consequently, the reduction in stratospheric ozone could lead to elevated incident UV radiation in the Red Sea region over the next few decades. An increase in UV would also likely lead to intensified photobleaching of CDOM, which in turn would further increase the optical transparency of the water column 13, 99. Additionally, SST of the Red Sea is increasing at a rate that exceeds the warming rate of the global ocean 53. This trend could affect the UV attenuation properties of the Red Sea; an increase in SST would result in stronger thermal stratification of the water column during summer and, potentially, deeper penetration of UV into the water column.

In summary, we found that UV attenuation in the Red Sea exhibits a distinct latitudinal gradient and that the basin contains some of the most optically transparent marine waters in the world. However, great uncertainty remains regarding future changes to UV attenuation and UV doses in the Red Sea as a result of imminent environmental change, and global warming and stratospheric ozone depletion in particular.
